# Coexistence of anti-SOX1 and anti-GABAB receptor antibodies with paraneoplastic limbic encephalitis presenting with seizures and memory impairment in small cell lung cancer: A case report

**DOI:** 10.3389/fimmu.2022.955170

**Published:** 2022-07-28

**Authors:** Sizhu Gong, Yue Han, Enling He, Min Liu, Xiyao Fu, Fang Deng

**Affiliations:** Department of Neurology, First Affiliated Hospital of Jilin University, Changchun, China

**Keywords:** anti-SOX1 antibody, anti-GABAB receptor antibody, small cell lung cancer, paraneoplastic limbic encephalitis, autoimmune encephalitis

## Abstract

**Purpose:**

Paraneoplastic neurological syndromes associated with autoantibodies are rare diseases that cause abnormal manifestations of the nervous system. Early diagnosis of paraneoplastic neurological syndromes paves the way for prompt and efficient therapy.

**Case report:**

we reported a 56-year-old man presenting with seizures and rapidly progressive cognitive impairment diagnosed as paraneoplastic limbic encephalitis (PLE) with anti-SRY-like high-mobility group box-1 (SOX-1) and anti-γ-aminobutyric acid B (GABAB) receptor antibodies and finally confirmed by biopsy as small cell lung cancer (SCLC). At the first admission, brain magnetic resonance imaging (MRI) showed no abnormal signal in bilateral hippocampal regions and no abnormal enhancement of enhanced scan. The serum anti-GABAB receptor antibody was 1:100 and was diagnosed as autoimmune encephalitis (AE). The computed tomography (CT) scans of the chest showed no obvious tumor signs for the first time. Although positron emission tomography-computed tomography (PET-CT) revealed hypermetabolism in the para mid-esophageal, the patient and his family declined to undertake a biopsy. The patient improved after receiving immunoglobulin, antiepileptic therapy, and intravenous methylprednisolone (IVMP) pulse treatment. However, after 4 months, the symptoms reappeared. Brain MRI revealed abnormal signals in the hippocampal regions. Reexamination of the cerebral fluid revealed anti-GABAB receptor and anti-SOX-1 antibodies, which contributed to the diagnosis of PLE. SCLC was found in a para mid-esophageal pathological biopsy. Antiepileptic medications and immunoglobulin were used to treat the patient, and the symptoms were under control.

**Conclusion:**

Our findings increase the awareness that patients with limbic encephalitis with cognitive dysfunction and epileptic seizures should be enhanced to detect latent malignancy. Our case also highlights the importance of anti-SOX1 antibodies in the detection of underlying neoplasm, particularly SCLC. Our findings raise awareness of the cognitive impairment seen by patients with limbic encephalitis.

## Introduction

Paraneoplastic neurological syndromes (PNSs) are a group of clinical heterogeneous syndromes associated with cancer involving the nervous system (1), including the limbic system, cerebellum, peripheral nervous symptoms, and neuromuscular junction (2). Classic PNS includes Lambert-Eaton myalgia syndrome (LEMS), paraneoplastic cerebellar degeneration (PCD), paraneoplastic limbic encephalitis (PLE), encephalomyelitis, and sensory neuronopathy (2). PLE is an inflammatory process that involves structures at the limbic system in the brain and is characterized by frequent epileptic seizures, mental behavioral disorders, and memory and cognitive deficits, and is a rare paraneoplastic syndrome (3). The mechanism is the cross-reactivity of antibodies to ectopic antigens expressed by tumor cells with antigens of the nervous system, thus initiating an immune and allied response selectively affecting the limbic system. Approximately 50% of patients with anti-GABAB receptor (GABABR) antibodies develop underlying tumors, most frequently small cell lung cancer, and present with limbic encephalitis (LE) with prominent seizures (4). SOX-1 is a member of the SOX protein family, and anti-SOX-1 antibodies may serve as a sensitive predictor of PNS and contributes to an early diagnosis of underlying tumors (5, 6).

Here, we reported a 56-year-old man presenting with seizures and memory dysfunction diagnosed as PLE with anti-SOX-1 and anti-GABABR antibody and finally confirmed by biopsy as small cell lung cancer (SCLC).

## Case presentation

**Figure 1:**
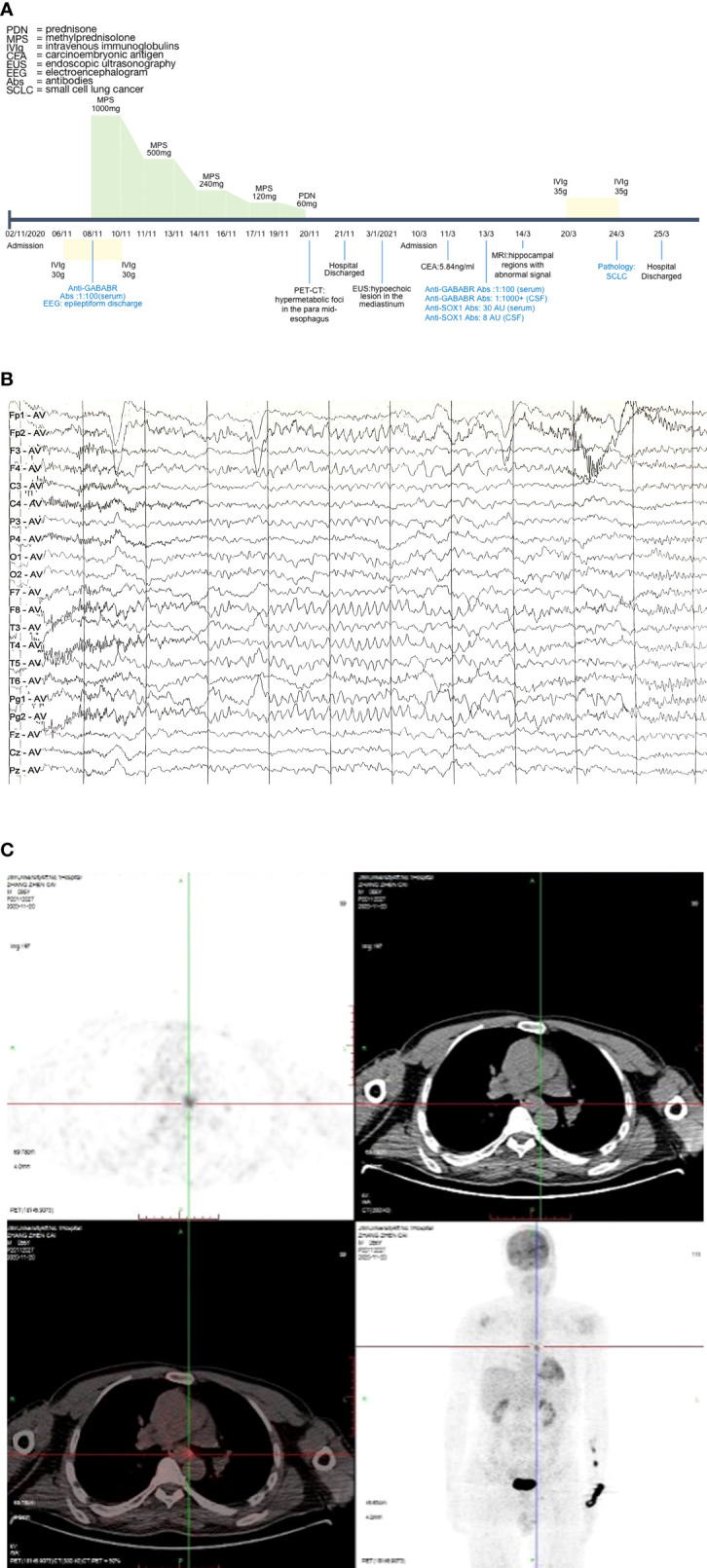
Case progress timeline, EEG, and PET-CT. **(A)** Case progress timeline and vital treatment records. **(B)** EEG demonstrated epileptiform discharge. **(C)** PET-CT suggested that the metabolism of para mid-esophageal was increased. The lesion was about 1.7 cm*0.6 cm, the maximum value of SUV was 4.4, and the lesion was poorly demarcated from the adjacent esophageal wall. EEG, electroencephalogram; PET-CT, positron emission tomography-computed tomography; SUV, standard uptake value.

A 56-year-old male patient presented to the hospital with an epileptic seizure, amnesia, slow reaction, and irritability. After drinking alcohol, he began experiencing epileptic seizures around 3 days ago, manifesting as consciousness disturbance, rolling eyes, foaming at the mouth, and body shaking with a tongue bite. His memory impairment began about a month ago, as evidenced by his failure to recall recent occurrences. He was examined at a local hospital and given antiepileptic medication. The seizure reappeared during the duration of therapy, and the body temperature increased to 38°C. Then the fever subsided, and he visited our hospital for further treatment. He experienced one convulsion during admission, lasting about 310 s, and it was the same as the first time. The patient had a lengthy history of drinking and smoking, consuming two packs every day. He was previously healthy and had no family history of psychiatric disorders or cognitive disorders. Physical examination on admission showed the following results: vital signs were normal, and blood pressure was 132/86 mmHg on admission. The cardiovascular, pulmonary, and abdominal examinations did not show abnormalities. His neurological physical examination revealed sanity and was remarkable for decreasing computational ability and short-term memory. There were no abnormalities in the cranial nerves, muscle tone, muscle power, deep tendon reflex, or coordination (timeline in **Figure 1A**).

### Investigation

The patient had normal levels of vitamins B and E, cholesterol, albumin, and ethanolamine as well as no kidney, liver, or thyroid impaired function. Glycated hemoglobin, basal glycemia levels, and serum tumor markers were within range. The cerebrospinal fluid (CSF) was translucent and had a clear appearance. The CSF pressure was 120 mmH_2_O (normal value: 80–180 mmH_2_O), while protein, erythrocytes, and leukocytes were within range. Pandy’s test showed positive results. A cytological preparation of cerebral spinal fluid revealed occasional lymphocytes and no abnormal cells. Cerebrospinal fluid immunoglobulin G (IgG) was 52.50 mg/l. Human immunodeficiency virus, cytomegalovirus, herpes simplex virus I/II DNA, toxoplasma gondii-DNA, and rubella virus RNA were undetectable in serum. The above results were from the central laboratory of the First Affiliated Hospital of Jilin University. Additionally, it should also be mentioned that the initial dilution gradients of serum and cerebrospinal fluid are 1:1 and 1:10, respectively. Cell-based assay (CBA) was utilized for the detection of antibodies for autoimmune encephalitis, while BLOT was used for the detection of antibodies for paraneoplastic disease. The anti-GABABR antibody IgG was positive (1:100) in the serum. However, the biomarkers of paraneoplastic syndrome antibodies (anti-Hu, -Yo, -Ri, -CV2, -Amphiphysin, -Ma, -SOX1, -DNER, -Zic4, -GAD65, -PKC, -Recoverin, -MGT30) and the rest of autoimmune encephalitis antibodies (NMDA, LGI1, CASPER2, AMPAR, IgLON5, mGluR5, GlyR5, D2R) were all negative in serum and CSF.

On neuropsychological assessment, the MMSE (Mini-Mental State Examination) score was 19/30 and the MES (Memory and Executive Screening) score was 45/100. The MoCA (Montreal Cognitive Assessment) score was 11/30. The scores of HIS (Hachinski Ischemia Scale) and ADL (Activity of Daily Living Scale) were 2/18 and 28/80, respectively. The CDR (Clinical Dementia Rating) score was 2, suggesting moderate dementia. Brain magnetic resonance imaging (MRI) showed bilateral multiple lacunar cerebral infarction, bilateral hippocampal regions were normal, and postcontrast enhancement revealed no obvious enhancement of lesions. The chest computed tomography (CT) scan showed no obvious tumor signs. Electroencephalogram (EEG) demonstrated epileptiform discharge (**Figure 1B**). Positron emission tomography-computed tomography (PET-CT) scan revealed hypermetabolic foci in the para mid-esophagus, which was poorly demarcated from the adjacent esophageal wall and measured 1.7 cm*0.6 cm (**Figure 1C**). Then, we recommended that the patient undergo a biopsy to exclude malignancy, but the patient and his family refused. Consequently, we were unable to do a biopsy in order to identify the tumor.

The final diagnosis was anti-GABABR encephalitis, status epilepticus, and rapidly progressive dementia. During admission, treatment was started with intravenous immunoglobulin (IVIg 0.4 g/kg*day for 5 days), antiepileptic treatment (sodium valproate 0.5 g b.i.d. and levetiracetam 0.25 g b.i.d. orally, and phenobarbital sodium 0.2g intramuscular q.d.), and intravenous methylprednisolone (IVMP) pulse therapy. He was initiated on IVMP pulse therapy (1,000 mg for 3 days). Then, he showed memory impairment improvement and IVMP pulse therapy was gradually tapered (500, 240, and 120 mg/day for 3 days each). After 12 days, prednisolone was taken orally at 60 mg/day for 7 days. The memory impairment improved, and there was no seizure recurrence. After discharge from the hospital, the patient took prednisolone orally (prednisolone was reduced by 5 mg every 7 days and until maintaining the therapeutic dose of 5 mg), sodium valproate 0.5 g b.i.d., and levetiracetam 0.25 g b.i.d. We conducted a telephone follow-up, and the results demonstrated that his seizures entirely recovered. The patient later underwent endoscopic ultrasonography (EUS) as an outpatient which revealed a hypoechoic lesion in the mediastinum, but the nature was to be investigated. However, the patient still did not undergo a puncture biopsy.

Four months later (13 March 2021), the patient presented to the hospital again with memory impairment for 20 days, which manifests as forgetting about his relatives. Additionally, he developed sleep dysfunction that showed difficulty in falling asleep at night and waking up easily. The abnormal examination on admission showed intermittent speech disorder and aprosexia. The tumor marker of carcinoembryonic antigen (CEA) increased, showing 5.84 ng/ml (normal value: <5 ng/ml). The cerebrospinal fluid appearance was transparent, and cerebrospinal fluid IgG was 50.97 mg/l. The CSF pressure, protein, erythrocytes, and leukocytes were within range. The anti-GABABR antibody was positive in both CSF and serum (cerebrospinal fluid anti-GABABR antibody IgG: 1:100+, serum anti-GABABR antibody IgG: 1:1,000+). Anti-SOX1 antibody IgG was 8 AU (arbitrary units) in CSF, and anti-SOX1 antibody IgG was 30 AU in serum (negative: <5 AU, positive: >10 AU. If the results are within 5–10 AU, rechecked after 8–12 weeks). Additionally, the rest of paraneoplastic syndrome antibodies (anti-Hu, -Yo, -Ri, -CV2, -Amphiphysin, -Ma, -DNER, -Zic4, -GAD65, -PKC, -Recoverin, -MGT30) and autoimmune encephalitis antibodies (NMDA, LGI1, CASPER2, AMPAR, IgLON5, mGluR5, GlyR5, D2R) were negative in both CSF and serum.

**Figure 2:**
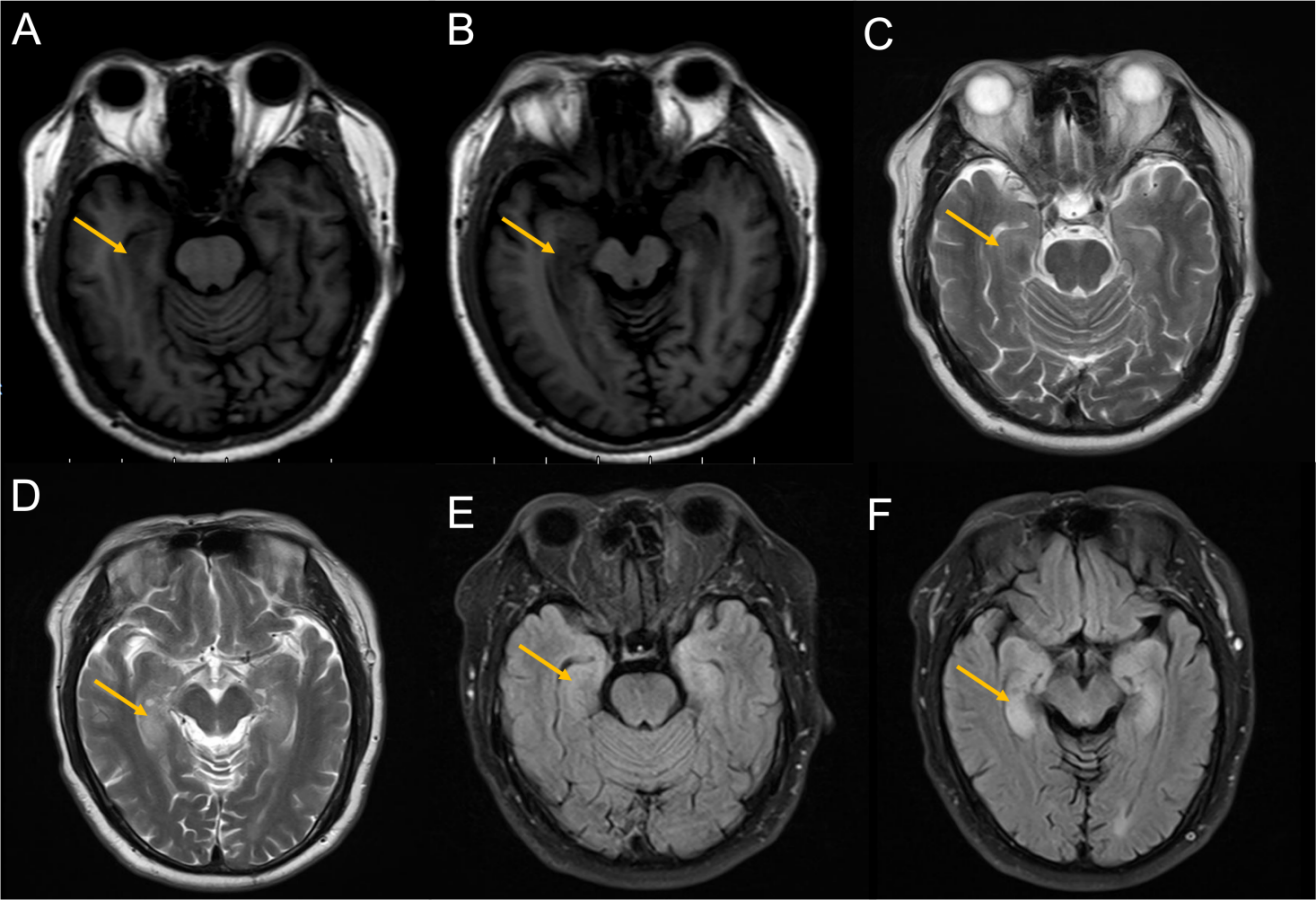
Images from the MRI after the second admission. **(A, B)** T1-weighted sequences showed slight hypointensity in the hippocampus (yellow arrow). **(C, D)** T2-weighted sequences showed hyperintensity in the hippocampus (yellow arrow). **(E, F)** FLAIR showed hyperintensity in the hippocampus (yellow arrow). magnetic resonance imaging; FLAIR, fluid-attenuated inversion recovery.

**Figure 3:**
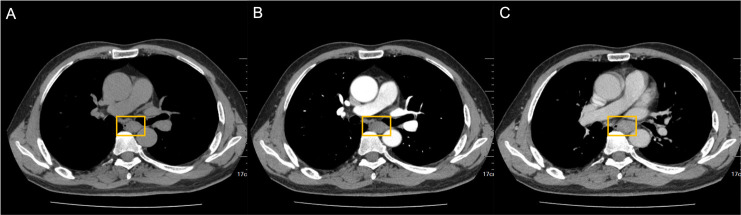
Paraesophageal space-occupying lesion visible on chest CT. nodular thickening of the horizontal tube wall of the esophagus from the tracheal carina was 2.7 cm*2.0 cm*4.0 cm (yellow square). **(A)** CT plain scan. **(B)** Arterial phase. **(C)** Venous phase. CT, computed tomography.

**Figure 4:**
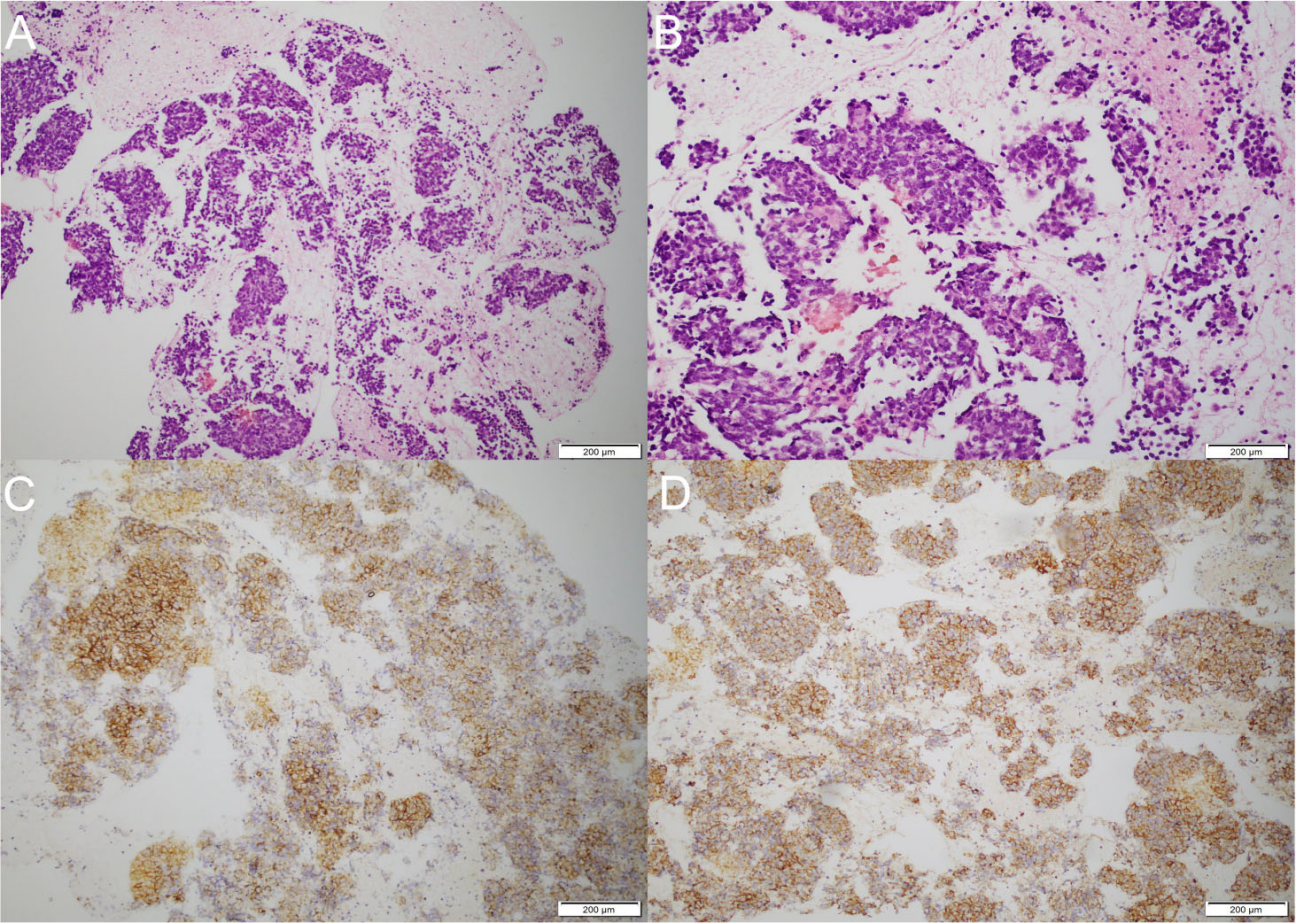
Pathological examination and immunohistochemistry. **(A)** Biopsy of paraesophageal lesion (hematoxylin–eosin staining, magnification ×40). **(B)** Biopsy of paraesophageal lesion (hematoxylin–eosin staining, magnification ×100). **(C)** The immunohistochemistry demonstrated positive results for synaptophysin. **(D)** The immunohistochemistry demonstrated positive results for CD56.

Brain MRI revealed bilateral hippocampal regions with abnormal signal, showing a slightly high signal in T2-weighted sequences and a slightly high signal shadow in fluid-attenuated inversion recovery (FLAIR) (**Figure 2**). The chest CT scan showed paraesophageal space-occupying lesion, considering the possibility of malignant lesions in the patient’s mediastinum (**Figure 3**). Fine needle aspiration (FNA) of his mediastinal lesions showed positive results for malignant cells. The immunohistochemistry showed positive results for TTF1, CK, synaptophysin, villin, CgA, E-cad, and CD56. CK7 and CEA scattered positively and showed a 70% ki67 nuclei-positive staining (**Figure 4**). The pathology report confirmed a poorly differentiated neuroendocrine carcinoma, suggestive of an SCLC.

### Differential diagnosis

Although it is important to consider the diagnosis of PLE in the patient with cognitive impairment, neuropsychiatric symptoms, and seizures, other causes that caused these neuronal and psychiatric symptoms should not be ignored. Patients with symptoms of neuropsychiatric dysfunction and seizure may also be attributable to viral encephalopathies (VE). Moreover, most patients present with non-specific symptoms including fever, headache, consciousness disturbance, and abnormal behaviors, and it is easy to have a misdiagnosis in clinical work. Considering his symptoms and that he had a fever before admission, we assumed he had VE. However, his laboratory examination revealed no alterations in parameters of the virus, such as human immunodeficiency virus, cytomegalovirus, herpes simplex virus I/II DNA, toxoplasma gondii-DNA, and rubella virus RNA. After excluding VE, we considered that potential PNSs might account for the seizure and memory impairment in a male smoker patient. Considering this, diagnosis hinges crucially on lumbar puncture examination of specific anti-neuronal antibodies in serum and CSF. Consequently, we chose to detect the anti-neuronal antibody spectrum and a PET-CT scan. The anti-GABABR antibody in serum was positive, which was associated with SCLC. PET-CT scan revealed that the metabolism of para mid-esophageal was increased, suspicious of malignancy, but the patient and his family refused to undergo a biopsy. After 4 months, anti-GABABR and anti-SOX1 antibodies were positive and brain MRI revealed a limbic system with an abnormal signal, which was a predictor for underlying malignancy, especially SCLC. After performing FNA of the mediastinal lesions, the pathology report confirmed a poorly differentiated neuroendocrine carcinoma, supporting the judgment of SCLC.

### Diagnosis and treatment

The final diagnosis showed (1) small cell lung cancer and (2) paraneoplastic limbic encephalitis (anti-SOX1 and anti-GABABR antibodies both positive).

Treatment: After administration of intravenous immunoglobulin (IVIg 0.4 g/kg*day for 5 days) and antiepileptic treatment (sodium valproate 0.5g b.i.d, and levetiracetam 0.25g b.i.d), his memory impairment and neuropsychiatric symptoms improved.

### Outcome and follow-up

The family decided to discontinue the anticancer therapy after careful thought. We conducted a telephone follow-up in July 2022, and the results demonstrated that while his psychiatric symptoms and seizures had subsided and his sleep disorders had entirely recovered, memory dysfunction—which manifests as forgetting about recent events—remained. He gave up using radiotherapy and chemotherapy to treat tumors in the case of SCLC, and Chinese medicine became a vital part of his tumor treatment. We hypothesized that the SCLC-associated immune response would suppress tumor development.

## Discussion

The patient was a middle-aged to elderly man with no respiratory symptoms and weight loss. The main neurological manifestations were seizures, short-term memory impairment, and abnormal psychiatric behavior, and imaging showed abnormal signals in the limbic system. Small cell lung cancer was diagnosed after the onset of neurological symptoms at 4 months. Based on the patient’s history, clinical manifestations, imaging, and antibody tests in blood and CSF, he was diagnosed with PLE and SCLC (7). In this case, it is believed that the anti-GABABR antibody has been associated with seizures, memory dysfunction, and behavioral abnormalities, whereas the anti-SOX-1 antibody may not be the primary antibody responsible for these symptoms but has a pointing significance for the diagnosis of PLE and the identification of cancer. Paraneoplastic neurological syndromes result from immune-mediated direct damage to the nervous system rather than direct invasion and metastasis of tumors (8). PNS may be the earliest manifestation of malignancy and frequently prior to the detection of tumor, but due to the lack of biomarkers, diagnosis of PNS is challenging (9).

With the development of the anti-neuronal antibody spectrum, anti-neuronal antibodies were divided into two types based on the target antigen location: intracellular (onconeuronal) antibodies and autoantibodies to neuronal cell-surface antigens (9). Anti-intracellular antibody-related disorders affect the elderly and have a positive tumor incidence of up to 95% and are also known as paraneoplastic syndrome antibodies, including anti-Hu, Yo, Ri, CV2, Amphiphysin, Ma, and SOX-1 antibodies. Anti-intracellular antibodies are directed against intracellular nuclear proteins and cause irreversible neuronal damage or apoptosis through cytotoxic T-cell-mediated immune responses. Even with some improvement in symptoms, there is no satisfactory response to treatment and with poor prognosis. Therefore, anti-onconeuronal antibodies that per se exert a direct pathogenic effect in PNS are unlikely. On the other hand, antibodies to anti-neuronal surface antigens are associated with autoimmune encephalitides, such as anti-NMDA, LGI1, GABABR, AMPAR, mGlu5, and CASPER2 antibodies. Autoimmune encephalitis (AE) patients respond well to immunotherapy compared with classical paraneoplastic disorders (10); when AE-related antibodies are removed, neuronal dysfunction is usually reversed.

GABABR is G protein-coupled receptor expressed in the presynaptic membrane and postsynaptic membrane and is distributed throughout the central nervous system and peripheral nervous system. GABA is widely expressed in the cerebral cortex, thalamus, cerebellum, amygdala, and hippocampus (11). Knockdown of the GABAB receptor gene in animal models resulted in memory behavior disorders, epilepsy, and nociceptive hypersensitivity (12), but the pathogenesis is not yet clear. It may be that the lack of normal functional structural GABAB receptors leads to recurrent seizures (13). The first case of anti-GABABR encephalitis was presented in 2010 by Lancaster et al., which revealed that 395 of 410 patients with suspected paraneoplastic or autoimmune encephalitis were positive for the antibody, and the target of the antibody was finally confirmed to be the GABAB receptor (14). The onset of the disease is usually in middle-aged and elderly men with an average age of 52 years, accompanied by early and prominent seizures, memory and cognitive deficits, and mental behavior disorders (15). Fifty percent of anti-GABABR-positive patients present with underlying cancer, most frequently SCLC, requiring long-term follow-up. In our case, the patient’s symptoms were consistent with those described above and SCLC was found after 4 months.

SOX-1 is a member of the SOX protein family and is identified as an important transcription factor for embryonic development, sharing a conserved DNA-binding protein. SOX-1 is selectively expressed in Bergman glial cells of the adult cerebellum cortex, involved in the repair of neural stem cells, development of the central nervous system, and downregulation in adults (5). SOX-1 protein is expressed in neuroendocrine tumors, such as small cell lung cancer (6). Additionally, SOX proteins affect the differentiation of airway epithelial cells and are highly immunogenic (8), which explains why the antibody can be found in SCLC. The protein encoded by SOX-1 is involved in the development of small cell lung cancer, and its expression is downregulated in tumor tissues (16), indicating that the pathogenesis may be closely associated with aberrant methylation of the SOX-1 gene promoter (17). Anti-SOX-1 antibodies are onconeuronal antibodies targeting a protein of the SRY-like high-mobility superfamily group in the Bergmann cell nucleus, which prevents neural differentiation in progenitor cells (18). Anti-SOX-1 antibodies are associated with a variety of neurological syndromes, of which LEMS is most commonly associated with anti-SOX-1 antibodies, followed by PCD (5). Although the specific pathogenesis of the association between anti-SOX-1 antibodies and the paraneoplastic syndrome is unclear, the predominant tumor in patients with anti-SOX-1 antibodies is SCLC. SCLC is a neuroendocrine-differentiated neoplasm that accounts for 15% of all lung cancers and is the most aggressive form of lung cancer (17) (19). Small cell lung cancer tumor cells are highly immunogenic and contain multiple neuronal antigens; patients with SCLC will develop neurological symptoms that precede the detection of the tumor (20). In addition, in a previous study that analyzed 489 patients with clinically suspected PNS, antibodies were found in both cerebrospinal fluid and blood in only 18 patients (21). In our case, antibodies were found in the patient’s blood and CSF. Although intrathecal synthetic antibodies are present in a few patients, the presence of autoantibodies is a predictor of tumor development.

These autoantibodies react with antigens expressed in tumor and neuronal structures, leading to the hypothesis of autoimmune pathogenesis (9). Although there is a lack of detailed studies to confirm a direct pathogenic role of anti-neuronal antibodies, the common consensus is that the presence of autoantibodies predicts tumorigenesis and points to a specific underlying tumor. There are various well-characterized onconeural antibodies associated with a specific type of cancer. PNS occurs in 1% to 3% of all cancer patients and is most common in lung cancer (especially small cell lung cancer), followed by lymphoma and gynecological tumors (6). Anti-Hu-positive patients have SCLC in about 87%, 86% of patients with anti-Ri have either lung or breast cancer, young patients with anti-Ma2 have the possibility to suffer from germ-cell tumor, and more than 90% of patients with anti-Yo-positive antibodies have breast or ovarian tumor (22) (23). Fifty percent of patients with anti-GABABR antibodies develop underlying tumors, most frequently small cell lung cancer, and present with limbic encephalitis with prominent seizures (4).

Antineuronal autoantibodies could be the main predictor of tumors in patients. PLE-induced neurological damage may be earlier than the clinical symptoms due to the primary tumor. Around 50% of PLE patients were negative for paraneoplastic antibodies, whereas the diagnosis of tumors was delayed by an average of nearly 7 months (20), with a maximum time from the onset of neurological symptoms to the final detection of the tumor of 5 years. The anti-SOX1 antibody positivity was 22% in SCLC alone and 32% in SCLC combined with PNS (6). This finding suggests that anti-SOX-1 antibodies may serve as a sensitive predictor of SCLC (6). Notably, patients with antibodies (SOX-1, Hu, Yo, Zic4, and CV2) but without tumors were not confirmed as SCLC until 30 months after disease onset (24). In a small group of 520 patients with anti-SOX-1 accompanying neurological symptoms, only 34 of 520 patients did not develop cancer even after a long-term follow-up (17). However, the possibility of patients having occult tumors cannot be ruled out, as most patients are not screened for cancer. Thereby, if PNS antibodies are found, a full-body scan such as chest CT, abdomen, breast, and pelvic examination should be performed. In addition, tumor growth may be limited because the immune system recognizes the tumor antigens as foreign and produces an immune response, so it is possible that the cancer is too small to have been detected or may develop in the future. So even if the associated antibodies are negative, the tumor cannot be excluded and screening for the primary tumor and long-term follow-up are still required.

With the improved understanding of the disease and the refinement of the antibodies’ profile, the coexistence of multiple antibodies in the same patient has attracted attention. The immune system suppresses tumor growth reflecting a cascade of immune system responses against tumor antigens, producing a variety of anti-neuronal antibodies (6). Anti-SOX-1 antibody and anti-GABABR antibody are markers of paraneoplastic syndrome, both of which are mostly seen in small cell lung cancer. Hoftberger et al. summarized that five of 20 patients with anti-GABABR encephalitis had small cell lung cancer, three of which were combined with positive anti-SOX-1 antibodies (25). The incidence of PNS-related antibodies and AE-related antibodies combined with tumors is as high as 95% (26), and the proportion of anti-GABABR antibodies combined with paraneoplastic-related antibodies is the highest. It may be related to tumor-induced anti-GABABR antibody production.

The coexistence of multiple antineuronal antibodies has a certain guiding significance and is closely associated with prognosis. Patients with additional paraneoplastic autoimmunity antibody coexistence may have more complex clinical features and may have a worse treatment outcome as a potential predictor of poor prognosis compared with the presence of a single antibody. Hardy-werben et al. reported that small cell lung cancer with the presence of antibodies alone had a better prognosis than antibodies coexisting (27). The presence of multiple autoantibodies coexisting with anti-SOX1 antibodies could be a stronger predictor of an SCLC diagnosis than the presence of only anti-SOX1 antibodies (28, 29). Notably, several studies have reported that the survival rate of SCLC and PLE patients is higher than in SCLC patients without PLE, and the presence of autoantibodies in small cell lung cancer predicts a good prognosis. Additionally, the presence and higher titers of anti-SOX1 antibodies seem to be associated with better therapeutic responses in patients with SCLC (30); perhaps because PNS is discovered earlier than cancer, autoantibodies provide a diagnostic time advantage that led to early diagnosis and treatment of cancer, thereby increasing the life expectancy of patients.

Strategies for PLE involved removing antibodies from circulation (IVIg, plasma replacement) and reducing the production of curative antibodies (large amounts of glucocorticoids, radiotherapy, and chemotherapy for tumors). Active treatment of the primary tumor can further prevent the damage of paraneoplastic antibodies to the nervous system, which is the basis of PNS treatment. Treatment against antibodies can only temporarily relieve the symptoms of the nervous system. Considering that small cell lung cancer has special pathological features, surgical treatment is not recommended. However, PLE depends heavily on the effectiveness of antitumor therapy, and only treatment against the primary tumor can improve the prognosis of patients (31).

## Conclusion

This review of the clinical features of a patient with anti-SOX-1 antibodies and anti-GABAB receptor antibodies has practical implications. First, encephalitis patients with impaired memory, abnormal mental behavior, and epileptic seizures should be determined for the presence of anti-neuronal autoimmune antibodies, which may be useful for detecting underlying malignancies. Second, PNS patients with anti-SOX-1 antibodies or additional anti-neuronal antibodies should be screened for tumors, particularly small cell lung cancer. Meanwhile, non-small cell lung cancer and tumors of other organs should be alerted. Third, PNS-related antibodies may serve as cancer predictors. Even patients who are currently positive for anti-SOX-1 antibodies but have not suffered from cancer still have the potential to develop cancer in the future. Thus, it is crucial to have a regular follow-up in order to maximize the timely detection of potential cancers. Fourth, it is important to apply oncologic therapy and immunotherapy immediately; although immunotherapy only provides temporary relief of neurological symptoms, the efficacy of immunotherapy needs to be further verified. Fifth, the higher malignancy of the primary tumor and coexistence of multiple paraneoplastic antibodies are closely associated with poor prognosis and are predictors of poor treatment outcomes.

## Data availability statement

The original contributions presented in the study are included in the article/supplementary material. Further inquiries can be directed to the corresponding author.

## Ethics statement

Written informed consent was obtained from the individual(s), and minor(s)’ legal guardian/next of kin, for the publication of any potentially identifiable images or data included in this article.

## Author contributions

SG performed information collection and drafted the manuscript. YH, EH, ML, and XF contributed to the literature review and manuscript preparation. FD supervised the review and approved the final version of the manuscript. All authors contributed to the article and approved the submitted version.

## Funding

This work was supported by a grant from the National Natural Science Foundation of China (82071293).

## Conflict of interest

The authors declare that the research was conducted in the absence of any commercial or financial relationships that could be construed as a potential conflict of interest.

## Publisher’s note

All claims expressed in this article are solely those of the authors and do not necessarily represent those of their affiliated organizations, or those of the publisher, the editors and the reviewers. Any product that may be evaluated in this article, or claim that may be made by its manufacturer, is not guaranteed or endorsed by the publisher.
